# 5-Hy­droxy-3-methyl-5-phenyl-4,5-di­hydro-1*H*-pyrazole-1-carbothio­amide

**DOI:** 10.1107/S1600536811036658

**Published:** 2011-09-30

**Authors:** Biplab Ganguly, Ana Foi, Fabio Doctorovich, Benu K. Dey, Tapashi G. Roy

**Affiliations:** a Department of Chemistry, University of Chittagong, Chittagong 4331, Bangladesh; b Departamento de Química Inorgánica, Analítica y Química, Física/INQUIMAE-CONICET, Facultad de Ciencias Exactas y Naturales, Universidad de Buenos Aires, Argentina

## Abstract

In the title compound C_11_H_13_N_3_OS, the aromatic ring and the dihydro­pyrazole ring are oriented orthogonally with respect to each other, making a dihedral angle of 89.92 (9)°. An intra­molecular O—H⋯S hydrogen bond occurs. In the crystal, weak N—H⋯N and N—H⋯S hydrogen bonds link the mol­ecules into a columnar stack propagating along the *b* axis.

## Related literature

For the biological activity of sulfur–nitro­gen ligand compounds, see: Wilder Smith (1964 ▶); Grii & Khare (1976 ▶); French & Blang (1966 ▶); Davis Parke & Co (1957 ▶); Vattum & Rao (1959 ▶); Brockaman *et al.* (1959 ▶). For the carcinostatics thio­semicarbazone-containing nitro­gen heterocycles, see: Freedlander & French (1958 ▶); French & Blang (1965 ▶).
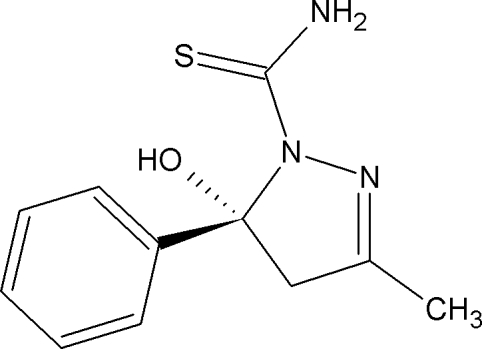


## Experimental

### 

#### Crystal data

C_11_H_13_N_3_OS
*M* *_r_* = 235.31
Monoclinic, 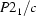

*a* = 11.6955 (6) Å
*b* = 7.6889 (4) Å
*c* = 13.7588 (10) Å
β = 111.978 (7)°
*V* = 1147.35 (12) Å^3^
*Z* = 4
Mo *K*α radiation
μ = 0.26 mm^−1^
*T* = 298 K
0.39 × 0.41 × 0.43 mm

#### Data collection

Oxford Diffraction Gemini E CCD diffractometer
Absorption correction: multi-scan (*CrysAlis PRO*; Oxford Diffraction, 2009 ▶) *T*_min_ = 0.541, *T*_max_ = 1.000
19392 measured reflections
2326 independent reflections
2161 reflections with *I* > 2σ(*I*)
*R*_int_ = 0.069

#### Refinement

*R*[*F* ^2^ > 2σ(*F* ^2^)] = 0.047
*wR*(*F* ^2^) = 0.130
*S* = 1.07
2326 reflections
157 parameters
H atoms treated by a mixture of independent and constrained refinement
Δρ_max_ = 0.27 e Å^−3^
Δρ_min_ = −0.35 e Å^−3^

### 

Data collection: *CrysAlis PRO* (Oxford Diffraction, 2009 ▶); cell refinement: *CrysAlis PRO*; data reduction: *CrysAlis PRO*; program(s) used to solve structure: *SHELXS86* (Sheldrick, 2008 ▶); program(s) used to refine structure: *SHELXL97* (Sheldrick, 2008 ▶); molecular graphics: *ORTEP-3 for Windows* (Farrugia, 1999 ▶); software used to prepare material for publication: *WinGX* publication routines (Farrugia, 1999 ▶).

## Supplementary Material

Crystal structure: contains datablock(s) I, global. DOI: 10.1107/S1600536811036658/ds2131sup1.cif

Structure factors: contains datablock(s) I. DOI: 10.1107/S1600536811036658/ds2131Isup2.hkl

Supplementary material file. DOI: 10.1107/S1600536811036658/ds2131Isup3.cml

Additional supplementary materials:  crystallographic information; 3D view; checkCIF report

## Figures and Tables

**Table 1:** Hydrogen-bond geometry (Å, °)

*D*—H⋯*A*	*D*—H	H⋯*A*	*D*⋯*A*	*D*—H⋯*A*
O1—H1*O*⋯S1	0.93 (3)	2.35 (3)	3.1256 (15)	141 (2)
N3—H3*A*⋯S1^i^	0.89 (2)	2.81 (2)	3.5827 (17)	145.9 (19)
N3—H3*B*⋯N1^ii^	0.87 (2)	2.30 (2)	3.158 (2)	168 (2)

Symmetry codes: (i) 
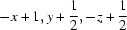
; (ii) 
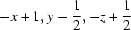
.

**Fig. 1.**
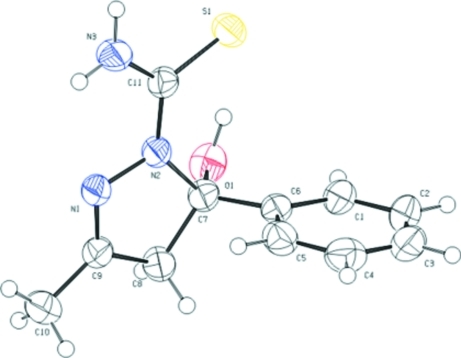
: ORTEP (50% probablity) diagram of the title compound.

**Fig. 2.**
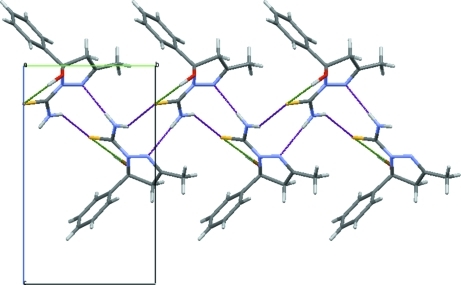
: Packing along a, showing the chain-like subunit formed. Intramolecular hydrogen bonds are displayed in green, and intermolecular hydrogen bonds in purple.

**Fig. 3.**
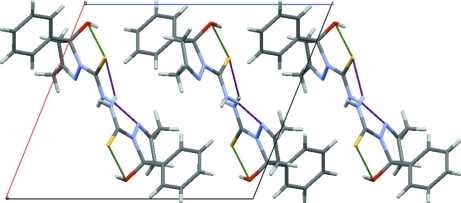
: Packing along b, showing the columnar arrangement of subunits. Intramolecular hydrogen bonds are displayed in green, and intermolecular hydrogen bonds in purple.

## supplementary crystallographic information

### Comment

Sulfur-Nitrogen ligand and their metal complexes have been reported as biologically important compounds possessing antiviral (Davis *et al.*, 1957), antibacterial (Vattum & Rao, 1959), antipyretic (Wilder Smith, 1964), fungicidal (Grii & Khare, 1976) and analgesic (Wilder Smith, 1964) activities. It was reported that pyridine-2carboxaldehyde dithiosemicarbazone displays anticancer activity. However, no mechanism of action was proposed (Brockaman *et al.*, 1959). French and co-workers (French & Blang, 1965; Freedlander & French, 1958; French & Blang, 1966) studied the carcinostatics thiosemicarbazones containing nitrogen heterocycles. The present investigation is an attempt to prepare a Schiff base ligand (HL) by the condensation of benzoyl acetone and thiosemicarbazide. During crystallization from ethanol-petroleum ether, the crystals of the title compound appropriate for single crystal X-ray diffraction were obtained.

In the crystal structure, the aromatic ring and the dihydropyrazole ring are oriented orthogonally with respect to each other [angle between these two rings is 89.92 (9) °]. Weak N–H···N (3.158 (2) Å) and N–H···S (3.5827 (17) Å) make the moloecules pack into a columnar stack propagating along *b* axis (see Figure 3).

### Experimental

Thiosemicarbazide purchased from the local market was crystallized from ethanol and dried under vacuum desiccator over silica gel (m.p. 441- 443 K) before use. A hot solution of benzoyl acetone (1.62 g, 10 mmol) in absolute ethanol was mixed with the hot solution of thiosemicarbazide (1.22 g, 10 mmol) in the same solvent. The mixture was refluxed for 6 h on a water bath. After reducing the volume, a white product was filtered off. This product was washed with ethanol for several times and dried in a vacuum desiccator over silica gel (m.p. 449–451 K. Yield 1.95 g (82.9%). Anal. Calc. for C_11_H_13_N_3_OS: C, 56.15; H, 5.57; N, 17.86; S, 13.62%. Found: C, 56.03; H, 5.61; N, 17.82; S, 13.57%. FT—IR (KBr, cm^-1^) νmax: 3360 (m, OH), 3260 (s, NH), 1642 (m, C=N), 999 (m, N—N). Then the crystals suitable for the crystallographic study were prepared by slow evaporation from a ethanol-petroleum ether (2:1 *v*/*v*) solution of the ligand.

### Refinement

Methyl groups were idealized (C—H = 0.96 A °) and allowed to ride. In all cases, H-atom displacement parameters were taken as *U*_iso_(H) = 1.5Ueq(C) for methyl groups or 1.2Ueq(C,*O*,*N*) otherwise.

### Crystal data

**Table:**

C_11_H_13_N_3_OS	*F*(000) = 496
*M**_r_* = 235.31	*D*_x_ = 1.362 Mg m^−^^3^
Monoclinic, *P*2_1_/*c*	Mo *K*α radiation, λ = 0.71073 Å
Hall symbol: -P 2ybc	Cell parameters from 10287 reflections
*a* = 11.6955 (6) Å	θ = 3.5–73.8°
*b* = 7.6889 (4) Å	µ = 0.26 mm^−^^1^
*c* = 13.7588 (10) Å	*T* = 298 K
β = 111.978 (7)°	Prism, white
*V* = 1147.35 (12) Å^3^	0.43 × 0.41 × 0.39 mm
*Z* = 4	

### Data collection

**Table:**

Oxford Diffraction Gemini E CCD diffractometer	2161 reflections with *I* > 2σ(*I*)
graphite	*R*_int_ = 0.069
ω scans, thick slices	θ_max_ = 26.3°, θ_min_ = 1.9°
Absorption correction: multi-scan (*CrysAlis PRO*; Oxford Diffraction, 2009)	*h* = −14→14
*T*_min_ = 0.541, *T*_max_ = 1.000	*k* = −9→9
19392 measured reflections	*l* = −17→16
2326 independent reflections	

### Refinement

**Table:**

Refinement on *F*^2^	Primary atom site location: structure-invariant direct methods
Least-squares matrix: full	Secondary atom site location: difference Fourier map
*R*[*F*^2^ > 2σ(*F*^2^)] = 0.047	Hydrogen site location: inferred from neighbouring sites
*wR*(*F*^2^) = 0.130	H atoms treated by a mixture of independent and constrained refinement
*S* = 1.07	*w* = 1/[σ^2^(*F*_o_^2^) + (0.0808*P*)^2^ + 0.2816*P*] where *P* = (*F*_o_^2^ + 2*F*_c_^2^)/3
2326 reflections	(Δ/σ)_max_ < 0.001
157 parameters	Δρ_max_ = 0.27 e Å^−^^3^
0 restraints	Δρ_min_ = −0.35 e Å^−^^3^

### Special details

**Table:**

Geometry. All e.s.d.'s (except the e.s.d. in the dihedral angle between two l.s. planes) are estimated using the full covariance matrix. The cell e.s.d.'s are taken into account individually in the estimation of e.s.d.'s in distances, angles and torsion angles; correlations between e.s.d.'s in cell parameters are only used when they are defined by crystal symmetry. An approximate (isotropic) treatment of cell e.s.d.'s is used for estimating e.s.d.'s involving l.s. planes.
Refinement. Refinement of *F*^2^ against ALL reflections. The weighted *R*-factor *wR* and goodness of fit *S* are based on *F*^2^, conventional *R*-factors *R* are based on *F*, with *F* set to zero for negative *F*^2^. The threshold expression of *F*^2^ > σ(*F*^2^) is used only for calculating *R*-factors(gt) *etc*. and is not relevant to the choice of reflections for refinement. *R*-factors based on *F*^2^ are statistically about twice as large as those based on *F*, and *R*- factors based on ALL data will be even larger.

### Fractional atomic coordinates and isotropic or equivalent isotropic displacement parameters (Å^2^)

**Table:**

	*x*	*y*	*z*	*U*_iso_*/*U*_eq_	
N1	0.38340 (12)	0.44242 (18)	0.08551 (10)	0.0382 (3)	
O1	0.09679 (11)	0.29274 (18)	0.04417 (11)	0.0503 (3)	
N3	0.47610 (13)	0.1959 (2)	0.22717 (13)	0.0479 (4)	
C11	0.36034 (14)	0.1729 (2)	0.16030 (12)	0.0370 (3)	
C10	0.36242 (19)	0.7124 (2)	−0.00962 (16)	0.0541 (5)	
H10C	0.4469	0.7273	0.0363	0.081*	
H10A	0.3138	0.8058	0.0008	0.081*	
H10B	0.3568	0.7128	−0.081	0.081*	
N2	0.31423 (12)	0.29247 (17)	0.08476 (10)	0.0392 (3)	
H3B	0.504 (2)	0.124 (3)	0.2795 (18)	0.054 (6)*	
H3A	0.521 (2)	0.286 (3)	0.2218 (18)	0.060 (6)*	
H1O	0.114 (2)	0.194 (4)	0.086 (2)	0.068 (7)*	
S1	0.27587 (4)	0.00137 (5)	0.17302 (3)	0.04557 (19)	
C6	0.17249 (13)	0.1559 (2)	−0.08034 (12)	0.0379 (3)	
C9	0.31586 (15)	0.5446 (2)	0.01356 (12)	0.0396 (4)	
C7	0.18692 (14)	0.2977 (2)	0.00036 (13)	0.0399 (4)	
C8	0.18774 (17)	0.4804 (2)	−0.04428 (16)	0.0489 (4)	
H8A	0.1694	0.4756	−0.1191	0.059*	
H8B	0.1278	0.5551	−0.0317	0.059*	
C1	0.07051 (15)	0.0470 (2)	−0.11386 (13)	0.0444 (4)	
H2	0.0119	0.0557	−0.0835	0.053*	
C3	0.1390 (2)	−0.0863 (3)	−0.23965 (14)	0.0559 (5)	
H4	0.1277	−0.1666	−0.293	0.067*	
C5	0.25740 (16)	0.1420 (2)	−0.12829 (14)	0.0476 (4)	
H6	0.3263	0.214	−0.107	0.057*	
C2	0.05503 (17)	−0.0744 (3)	−0.19207 (14)	0.0527 (5)	
H3	−0.0128	−0.1484	−0.2125	0.063*	
C4	0.2406 (2)	0.0223 (3)	−0.20755 (17)	0.0557 (5)	
H5	0.298	0.0149	−0.2394	0.067*	

### Atomic displacement parameters (Å^2^)

**Table:**

	*U*^11^	*U*^22^	*U*^33^	*U*^12^	*U*^13^	*U*^23^
N1	0.0370 (7)	0.0349 (7)	0.0363 (7)	−0.0034 (5)	0.0064 (5)	0.0002 (5)
O1	0.0396 (6)	0.0545 (8)	0.0526 (7)	0.0057 (5)	0.0122 (5)	−0.0049 (6)
N3	0.0386 (7)	0.0435 (8)	0.0461 (8)	0.0001 (6)	−0.0018 (6)	0.0117 (6)
C11	0.0363 (7)	0.0343 (7)	0.0348 (7)	0.0028 (6)	0.0068 (6)	−0.0003 (6)
C10	0.0601 (11)	0.0424 (9)	0.0524 (10)	−0.0035 (8)	0.0127 (9)	0.0093 (8)
N2	0.0341 (6)	0.0336 (7)	0.0376 (7)	−0.0028 (5)	−0.0007 (5)	0.0029 (5)
S1	0.0429 (3)	0.0374 (3)	0.0500 (3)	−0.00315 (15)	0.0100 (2)	0.00632 (16)
C6	0.0337 (7)	0.0356 (7)	0.0349 (8)	0.0001 (6)	0.0021 (6)	0.0054 (6)
C9	0.0431 (8)	0.0352 (7)	0.0344 (8)	0.0013 (6)	0.0077 (6)	−0.0005 (6)
C7	0.0318 (7)	0.0365 (8)	0.0398 (8)	0.0019 (6)	0.0002 (6)	0.0022 (6)
C8	0.0436 (9)	0.0357 (8)	0.0491 (10)	0.0028 (6)	−0.0036 (8)	0.0045 (7)
C1	0.0366 (8)	0.0482 (9)	0.0399 (9)	−0.0050 (7)	0.0046 (6)	0.0004 (7)
C3	0.0708 (12)	0.0495 (10)	0.0395 (9)	0.0022 (9)	0.0116 (8)	−0.0033 (8)
C5	0.0452 (8)	0.0446 (9)	0.0511 (10)	−0.0049 (7)	0.0161 (7)	0.0040 (8)
C2	0.0511 (9)	0.0516 (10)	0.0428 (9)	−0.0108 (8)	0.0031 (7)	−0.0042 (8)
C4	0.0659 (12)	0.0559 (11)	0.0502 (10)	0.0065 (9)	0.0273 (10)	0.0070 (8)

### Geometric parameters (Å, °)

**Table:**

N1—C9	1.279 (2)	C6—C5	1.387 (2)
N1—N2	1.4062 (18)	C6—C7	1.520 (2)
O1—C7	1.397 (2)	C9—C8	1.493 (2)
O1—H1O	0.93 (3)	C7—C8	1.535 (2)
N3—C11	1.334 (2)	C8—H8A	0.97
N3—H3B	0.87 (2)	C8—H8B	0.97
N3—H3A	0.89 (3)	C1—C2	1.384 (3)
C11—N2	1.340 (2)	C1—H2	0.93
C11—S1	1.6958 (16)	C3—C2	1.373 (3)
C10—C9	1.481 (2)	C3—C4	1.382 (3)
C10—H10C	0.96	C3—H4	0.93
C10—H10A	0.96	C5—C4	1.384 (3)
C10—H10B	0.96	C5—H6	0.93
N2—C7	1.5077 (18)	C2—H3	0.93
C6—C1	1.387 (2)	C4—H5	0.93
			
C9—N1—N2	108.12 (12)	N2—C7—C6	110.55 (12)
C7—O1—H1O	105.7 (15)	O1—C7—C8	108.45 (14)
C11—N3—H3B	117.4 (14)	N2—C7—C8	100.40 (12)
C11—N3—H3A	121.5 (15)	C6—C7—C8	112.34 (14)
H3B—N3—H3A	121 (2)	C9—C8—C7	104.14 (13)
N3—C11—N2	116.99 (15)	C9—C8—H8A	110.9
N3—C11—S1	120.81 (13)	C7—C8—H8A	110.9
N2—C11—S1	122.18 (11)	C9—C8—H8B	110.9
C9—C10—H10C	109.5	C7—C8—H8B	110.9
C9—C10—H10A	109.5	H8A—C8—H8B	108.9
H10C—C10—H10A	109.5	C2—C1—C6	120.76 (17)
C9—C10—H10B	109.5	C2—C1—H2	119.6
H10C—C10—H10B	109.5	C6—C1—H2	119.6
H10A—C10—H10B	109.5	C2—C3—C4	119.37 (18)
C11—N2—N1	119.56 (12)	C2—C3—H4	120.3
C11—N2—C7	127.61 (13)	C4—C3—H4	120.3
N1—N2—C7	112.54 (12)	C4—C5—C6	120.67 (17)
C1—C6—C5	118.38 (16)	C4—C5—H6	119.7
C1—C6—C7	121.50 (15)	C6—C5—H6	119.7
C5—C6—C7	119.93 (14)	C3—C2—C1	120.45 (17)
N1—C9—C10	122.10 (15)	C3—C2—H3	119.8
N1—C9—C8	114.56 (15)	C1—C2—H3	119.8
C10—C9—C8	123.34 (15)	C3—C4—C5	120.35 (19)
O1—C7—N2	110.74 (13)	C3—C4—H5	119.8
O1—C7—C6	113.58 (13)	C5—C4—H5	119.8
			
N3—C11—N2—N1	−6.3 (2)	C5—C6—C7—N2	−53.08 (19)
S1—C11—N2—N1	172.51 (12)	C1—C6—C7—C8	−116.66 (17)
N3—C11—N2—C7	−179.61 (16)	C5—C6—C7—C8	58.17 (19)
S1—C11—N2—C7	−0.8 (2)	N1—C9—C8—C7	−4.7 (2)
C9—N1—N2—C11	−173.10 (14)	C10—C9—C8—C7	175.24 (16)
C9—N1—N2—C7	1.16 (18)	O1—C7—C8—C9	120.77 (15)
N2—N1—C9—C10	−177.61 (15)	N2—C7—C8—C9	4.61 (18)
N2—N1—C9—C8	2.3 (2)	C6—C7—C8—C9	−112.86 (15)
C11—N2—C7—O1	55.5 (2)	C5—C6—C1—C2	1.1 (2)
N1—N2—C7—O1	−118.22 (15)	C7—C6—C1—C2	175.99 (15)
C11—N2—C7—C6	−71.3 (2)	C1—C6—C5—C4	−0.1 (3)
N1—N2—C7—C6	115.00 (14)	C7—C6—C5—C4	−175.07 (16)
C11—N2—C7—C8	169.90 (16)	C4—C3—C2—C1	1.1 (3)
N1—N2—C7—C8	−3.79 (18)	C6—C1—C2—C3	−1.6 (3)
C1—C6—C7—O1	6.9 (2)	C2—C3—C4—C5	−0.1 (3)
C5—C6—C7—O1	−178.28 (14)	C6—C5—C4—C3	−0.4 (3)
C1—C6—C7—N2	132.09 (15)		

### Hydrogen-bond geometry (Å, °)

**Table:**

*D*—H···*A*	*D*—H	H···*A*	*D*···*A*	*D*—H···*A*
O1—H1O···S1	0.93 (3)	2.35 (3)	3.1256 (15)	141 (2)
N3—H3A···S1^i^	0.89 (2)	2.81 (2)	3.5827 (17)	145.9 (19)
N3—H3B···N1^ii^	0.87 (2)	2.30 (2)	3.158 (2)	168 (2)

Symmetry codes: (i) −*x*+1, *y*+1/2, −*z*+1/2; (ii) −*x*+1, *y*−1/2, −*z*+1/2.
